# Computer-assisted Telephone Interview Techniques

**DOI:** 10.3201/eid1204.050756

**Published:** 2006-04

**Authors:** Martyn Kirk, Ingrid Tribe, Rod Givney, Jane Raupach, Russell Stafford

**Affiliations:** * OzFoodNet, Canberra, Australian Capital Territory, Australia;; † Communicable Disease Control Branch, Adelaide, South Australia, Australia;; ‡ OzFoodNet, Brisbane, Queensland, Australia

**Keywords:** case-control study, outbreak, investigation, control selection, epidemiology, CATIQ

**To the Editor:** Fox et al. used computer-assisted telephone interview (CATI) techniques in an outbreak of cryptosporidiosis (*1*). Australian health agencies have used CATI for several years. A case-control study during an outbreak of *Salmonella* Mbandaka in 1996 employed CATI to interview 15 case-patients and 45 controls; contaminated peanut butter was implicated (*2*). Foodborne disease outbreaks are often geographically widespread and suited to using CATI.

Australian health authorities investigate ≈100 outbreaks of foodborne disease each year, with 3–4 using CATI-based case-control studies. Some jurisdictions investigate outbreaks by using CATI interviews of controls sampled from a bank of potential study participants (*3*). Potential study participants are recruited at the conclusion of rolling risk factor survey interviews, similar to the Behavioral Risk Factor Surveillance System.

A "control bank" allows investigators to rapidly obtain contact details for appropriately matched controls because age and sex of all household members are recorded in a database. Using control banks with CATI allows completion of studies quicker than CATI or traditional methods alone (*4*). South Australia has used CATI during 11 case-control studies of salmonellosis, legionellosis, Q fever, campylobacteriosis, Shiga toxin–producing *Escherichia coli*, and cryptosporidiosis (http://www.dh.sa.gov.au/pehs/notifiable-diseases-summary/current-outbreak-table.htm).

During an Australian CATI survey about gastroenteritis, 5,123 (84%) of 6,087 households agreed to be in a control bank (*5*). This bank of 14,024 potential controls was used in 4 case-control studies of sporadic salmonellosis and campylobacteriosis. This system avoided randomly dialing thousands of households to enroll controls in young age groups. The control bank was used for 3 years after initial collection, although many jurisdictions update banks annually.

Investigators may find CATI useful, although it can be costly and introduce biases (*4*). Programming questionnaires can delay investigations, which makes paper-based collection better in small outbreaks (*4*). CATI cannot be used in areas where a small proportion of the population has telephones. Despite limitations, CATI, when combined with control banks, may improve outbreak investigations.
